# Treatment decisions and the use of MEK inhibitors for children with neurofibromatosis type 1-related plexiform neurofibromas

**DOI:** 10.1186/s12885-023-10996-y

**Published:** 2023-06-16

**Authors:** Amy E. Armstrong, Allan J. Belzberg, John R. Crawford, Angela C. Hirbe, Zhihong J. Wang

**Affiliations:** 1 Division of Pediatric Hematology/Oncology, Washington University School of Medicine, St. Louis, MO USA; 2 Department of Neurosurgery, Johns Hopkins University School of Medicine, Baltimore, MD USA; 3 CHOC Neuroscience Institute, Children’s Hospital of Orange County, Orange, CA USA; 4 Department of Pediatrics, Division of Child Neurology University of California Irvine, Orange, CA USA; 5 Department of Internal Medicine, Washington University School of Medicine, St. Louis, MO USA; 6 Division of Hematology and Oncology, Children’s Hospital of Richmond, Virginia Commonwealth University, Richmond, VA USA

**Keywords:** Neurofibromatosis type 1, Plexiform neurofibroma, MEK inhibitors, Surgery, Clinical decision making

## Abstract

Neurofibromatosis type 1 (NF1), the most common tumor predisposition syndrome, occurs when *NF1* gene variants result in loss of neurofibromin, a negative regulator of RAS activity. Plexiform neurofibromas (PN) are peripheral nerve sheath tumors that develop in patients with NF1 and are associated with substantial morbidity and for which, until recently, the only treatment was surgical resection. However, surgery carries several risks and a proportion of PN are considered inoperable. Understanding the genetic underpinnings of PN led to the investigation of targeted therapies as medical treatment options, and the MEK1/2 inhibitor selumetinib has shown promising efficacy in pediatric patients with NF1 and symptomatic, inoperable PN. In a phase I/II trial, most children (approximately 70%) achieved reduction in tumor volume accompanied by improvements in patient-reported outcomes (decreased tumor-related pain and improvements in quality of life, strength, and range of motion). Selumetinib is currently the only licensed medical therapy indicated for use in pediatric patients with symptomatic, inoperable NF1-PN, with approval based on the results of this pivotal clinical study. Several other MEK inhibitors (binimetinib, mirdametinib, trametinib) and the tyrosine kinase inhibitor cabozantinib are also being investigated as medical therapies for NF1-PN. Careful consideration of multiple aspects of both disease and treatments is vital to reduce morbidity and improve outcomes in patients with this complex and heterogeneous disease, and clinicians should be fully aware of the risks and benefits of available treatments. There is no single treatment pathway for patients with NF1-PN; surgery, watchful waiting, and/or medical treatment are options. Treatment should be individualized based on recommendations from a multidisciplinary team, considering the size and location of PN, effects on adjacent tissues, and patient and family preferences. This review outlines the treatment strategies currently available for patients with NF1-PN and the evidence supporting the use of MEK inhibitors, and discusses key considerations in clinical decision-making.

## Background

Neurofibromatosis type 1 (NF1) is an autosomal-dominant genetic disorder, which, although rare, is the most common tumor predisposition syndrome; its prevalence is estimated to range from approximately 1:2000 to 1:6000 [1–4]. NF1 is caused by pathogenic variants in the *NF1* gene that result in loss of functional neurofibromin, a negative regulator of RAS activity [5]. This results in constitutive activation of the RAS/RAF/MEK/ERK pathway, which is implicated in cell proliferation and survival and is central to driving tumor growth and progression [6, 7].

NF1 has a highly variable clinical presentation; severity and manifestations vary greatly even among people who carry the same genetic defect [8]. NF1 is mainly characterized by the presence of pigmented lesions, such as café-au-lait macules and skinfold freckling, and multiple neurofibromas, including plexiform neurofibromas (PN) [1, 9]. Morbidities affecting individuals with NF1 also include skeletal, ocular, and cardiovascular manifestations, neurodevelopmental disorders, and hormonal problems. In addition, health-related quality of life (QoL) is impaired [10] and life expectancy reduced in people with NF1 [11, 12], with the mean age at death reported as 52.3 years in men and 51.9 years in women [13]. Diagnostic criteria for NF1 were recently revised to reflect developments in genetics, ophthalmology, dermatology, and neuroimaging (Table 1) [14].

**Table 1:** Revised 2021 diagnostic criteria for neurofibromatosis type 1 (NF1) [14]

Two or more of the following in an individual who does not have a parent with NF1^a^
Café-au-lait macules (≥6) ● >5 mm in diameter in pre-pubertal patients ● >15 mm in diameter in post-pubertal patients
Axillary or inguinal freckles
Neurofibromas (≥2 of any type) or one PN
Optic pathway glioma
Lisch nodules (≥2) or choroidal abnormalities (≥2)
A distinctive osseous lesion such as ● Sphenoid dysplasia ● Anterolateral bowing of the tibia ● Pseudoarthrosis of a long bone
A heterozygous pathogenic NF1 variant

^a^One or more of these criteria are required in an individual who has a parent with NF1

*NF1* Neurofibromatosis type 1, *PN* Plexiform neurofibroma

PN are histologically benign peripheral nerve sheath tumors that develop in up to 50% of people with NF1 [15]; they are typically congenital or manifest in early childhood, growing most rapidly in children under 5 years of age [16–18]. PN grow along the length of the nerve and are most frequently located in the trunk or extremities, and they have the potential to cause often debilitating manifestations and can be associated with substantial morbidity [19]. NF1-PN can cause pain, disfigurement, and motor dysfunction, and in some cases airway dysfunction, visual impairment, and bladder or bowel dysfunction [16, 20–24]. An ongoing natural history study has shown that most people with NF1-PN have at least one PN-associated morbidity, with the most common being pain, followed by disfigurement and motor dysfunction [19]. Defects in vision, airway, and bowel/bladder function were also observed. Symptomatic PN tend to be larger than asymptomatic PN, and PN-related morbidities tend to worsen over time in the absence of effective treatment [19, 25]. Additionally, NF1-PN carry an 8–13% lifetime risk of transformation to malignant peripheral nerve sheath tumors [26], which have a poor prognosis [27] and are the leading cause of death in people with NF1 [28].

Management of NF1-PN involves regular monitoring and, where appropriate, addressing the signs and symptoms. The goal of treatment is to improve or prevent PN-associated morbidity and, if treatment is indicated, selection of a surgical or medical management option should be based on rigorous clinical assessment, ideally with input from all members of a multidisciplinary team (MDT). The objective of this review is to describe the treatment strategies currently available for patients with NF1-PN and emerging evidence to support medical therapy, and to discuss key considerations in clinical decision making.

## Treatment options for NF1-PN

**Fig. 1:**
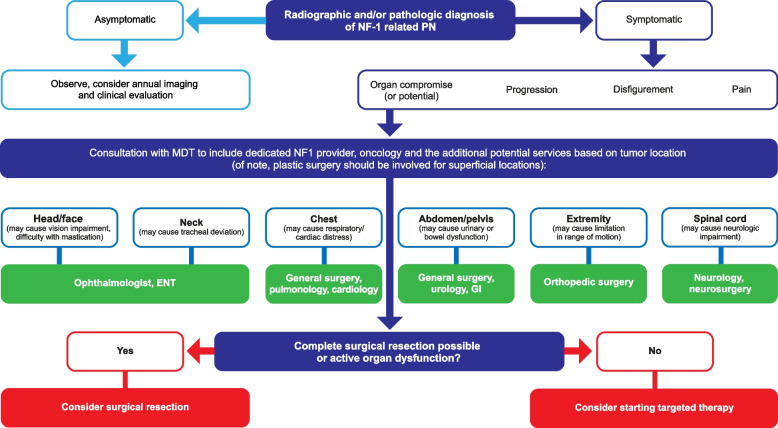
Multidisciplinary team management of patients with NF1-PN. *ENT* Ear nose and throat, *GI* gastrointestinal, *MDT* multidisciplinary team, *NF1* neurofibromatosis type 1, *PN* plexiform neurofibroma

Given the complex clinical presentation and treatment of NF1, an MDT may be beneficial, which can routinely include primary care providers, neurologists, geneticists, surgeons, neuropsychiatrists, and eye specialists (Fig. 1) [1, 29, 30]. As PN can arise from one or more nerves in any anatomic location, additional specialists may be included in the MDT for patients with more complex or unusual presentations. Given the risk of malignant transformation of NF1-PN, oncologists are becoming more involved in its management, particularly now that targeted therapies are available (Fig. 1).

Until recently, treatment options for NF1-PN have been limited to complete resection (surgical removal of all affected tissue) or debulking (partial surgical removal) as conventional chemotherapy or radiotherapy demonstrated limited clinical benefits [1, 30–32]. However, in many cases, surgical resection may not be possible due to PN location or size, and it is associated with a high risk of postoperative complications [30, 33, 34]. Historically, there have been considerable unmet treatment needs in NF1-PN, which have to some extent been addressed by recent developments in medical therapies.

### Surgery

Until recently, surgery was one of the only treatments available for NF1-PN and NF1-PN-related complications; it is still considered the only potentially curative treatment option [1, 5, 30, 32]. Its use and extent need to be tailored to the size of the PN, its location, growth rate, and radiologic features, and the overall general health and well-being of the patient [1, 30, 35]. Indications for surgery include neurologic compromise or impact on vital structures, pain, and disfigurement, with the overall aim of surgery to reduce morbidity and improve QoL [1, 30]. Approaches to surgical management of NF1-PN include complete resection and partial resection/debulking, whereby the goals of surgical management are to restore function, decrease pain, and improve PN-related disfigurement [36–39]. In many cases, this can only be partially achieved, by debulking, because complete resection is often not possible due to the size, location, and the network-like growth of PN [36–39]. Although surgical treatment is indicated for symptomatic NF1-PN, complete removal is frequently challenging because of the significant risks of bleeding and neurologic damage, especially in deep-seated PN involving multiple nerves [1, 30]. Approximately 50% of people with NF1-PN have PN that are considered inoperable, defined as those that cannot be completely resected without risk of substantial morbidity because of proximity to vital structures, invasiveness, or high vascularity [40]. Historically, treatment options to address inoperable PN have been limited to symptomatic management of morbidities that develop because of PN size and location. Therefore, further research is warranted to explore the potential use of medical therapy in these settings.

Collaborating surgeons in the MDT will develop a patient-specific surgical plan. As described above, this is tailored to the size and location of the PN, extent of neurologic involvement, vascularity of the PN, and possibility of malignancy [36]. Surgery may require subspecialty experience depending on tumor location, such as colorectal, orthopedic, ear nose and throat, and/or neurosurgery, with the aim of resection or debulking while preserving function. Plastic surgery may improve outcomes of surgical resection of smaller NF1-PN in superficial locations that might cause disfigurement, or be of assistance in wound closure. Clinical considerations regarding surgery are discussed in more detail in the clinical decision making section of this review [30, 41, 42].

### Medical therapy

In general, surgery remains the treatment of choice if the PN can readily be resected without significant morbidity. Unfortunately, surgery is challenging in many patients and medical therapy can be a useful option in symptomatic and inoperable NF1-PN [30]. Over the past two decades, the molecular basis of NF1-PN and the importance of the tumor microenvironment in the development of PN have been further elucidated [43–45]. This increased understanding provided the rationale for medical therapy directly targeting PN growth and development. A number of investigational agents have been evaluated in clinical trials for the treatment of PN [46]. Notably, imatinib, an inhibitor of KIT ligand that targets signaling between tumorigenic Schwann cells and mast cells within the tumor microenvironment, was the first medication that was shown to produce an objective response in patients with NF1-PN [44, 47]. More recently, inhibition of the dysregulated MAPK pathway has been shown to be a promising avenue for medical therapy [48, 49]. Several MEK inhibitors (selumetinib, mirdametinib, trametinib, binimetinib) and the tyrosine kinase inhibitor cabozantinib, which has activity against a broad range of targets, have been investigated in clinical trials in children and adults with NF1-PN (Table 2).

**Table 2:** Efficacy of MEK-inhibiting agents in clinical trials in patients with NF1-PN

Drug	Trial name and/or identifier (N)	Phase	Age, median (range), years	Baseline tumor volume, median (range), mL	Tumor shrinkage, median decrease from baseline (range), %	Patients with PR (≥ 20% decrease from baseline in PN volume by MRI)
Selumetinib	SPRINT NCT01362803^a^ (24)[49]	I	10.9 (3.0, 18.5)	1205 (29, 8744)	–31 (–47.0, –5.8)	17/24 (71%)
	SPRINT NCT01362803^b^ (50)[50]	II	10.2 (3.5, 17.4)	487 (5, 3820)	–27.9 (–55.1, 2.2)	34/50 (68%)
Mirdametinib	NCT02096471 (19)[51]	II	24 (16, 39)	364 (3.9, 5161)	–17.1 (–28.0, 48.7)	8/19 (42%)
	NCT03962543^c^ (20)[52]	II	33.5	Not reported	Not reported	7/20 (35%)
Cabozantinib	NCT02101736 (19)[53]	II	23 (16, 34)	557 (57, 2954)	–15.7 (–38.0, 2.8)	8/19 (42%)
Trametinib	NCT02124772 (26)[54]	I/IIa	5.5 (1, 16)	Not reported	Not reported	12/26 (46%)
Binimetinib	NCT03231306^d^ (25)[55]	II	23 (18, 55)	410 (7, 3128)	–26.5 (–21.1, –35.2)	13/20 (65%)

^a^Data cut-off January 4, 2016

^b^Data cut-off March 29, 2019

^c^ReNeu (NCT03962543) includes 50 patients aged 2–17 years and 50 patients aged ≥ 18 years; however, only data in 20 adults are available to date. An ORR of 50% was reported, with 6 or 7 PRs confirmed on subsequent assessment

^d^NCT03231306 includes patients aged ≥ 1 year; however, only data in adults are available to date

*MRI* magnetic resonance imaging, *NF1-PN* neurofibromatosis type 1-related plexiform neurofibroma, *PR* partial response

### MEK inhibitors approved for clinical use

#### Selumetinib

Selumetinib is an oral, selective, MEK1/2 inhibitor that is approved in the United States (US), the European Union (EU), and other countries for the treatment of pediatric patients (aged ≥ 2 years in the US and ≥ 3 years in the EU) with NF1 who have symptomatic, inoperable PN [56, 57]. Selumetinib has been investigated in a combined phase I/II clinical trial in children aged 2 to 18 years with NF1 and inoperable PN (SPRINT, NCT01362803). The SPRINT trial enrolled patients in two strata: stratum 1 for symptomatic PN with at least one PN-related complication and stratum 2 for PN with no clinically significant morbidity but with the potential for development of a PN-related complication. Volumetric MRI analysis was used to measure treatment response, with a ≥ 20% decrease in PN volume serving as the definition of a partial response (PR) in this trial and across all NF1-PN clinical trials [49, 50, 58]. 

In the phase I dose-finding part of the trial (data cut-off January 4, 2016), selumetinib treatment was associated with a sustained reduction in PN volume in the majority of patients, with confirmed PRs for ≥ 4 weeks reported in 17 of 24 children (71%; Table 2) [49]. PRs were sustained for a median of 23 28-day cycles (range: 6 to 42 cycles) [49]. In this phase I trial, selumetinib had acceptable rates of dose-limiting toxicity with a maximum tolerated dosage of 25 mg/m^2^ twice daily [49] (approximately 60% of the recommended fixed dose of 75 mg for adults [59]). In the phase II part of the trial (data cut-off March 29, 2019), most children with symptomatic PN (stratum 1) had durable tumor shrinkage and derived clinical benefit from selumetinib. A confirmed PR (defined as PR on consecutive restaging examinations at least 3 months apart) with 25 mg/m^2^ selumetinib twice daily was achieved by 34 of 50 patients (68%; Table 2), 28 of whom had a durable response lasting ≥ 1 year [50]. The median reduction in PN volume with selumetinib from baseline to best response was 27.9% (range: 2.2 to 55.1) [50]. Improvements in more than one patient-reported outcome were also achieved in 68% of selumetinib-treated patients after 1 year of treatment [50]. These included substantial decreases in PN-related pain, improvements in QoL, improvements in PN-related problems other than pain, improved strength, and clinically meaningful increases in range of motion [50].

A long-term analysis of SPRINT (data cut-off February 27, 2021) reported that nine patients in the phase I part of the trial remained on selumetinib, with a median treatment duration of 75.5 28-day cycles (range: 6 to 100) [60], and 23 patients in the phase II part of the trial remained on selumetinib after a median treatment duration of 55.5 28-day cycles (range: 1 to 73) [60]. Confirmed PR for the phase II part remained at 68% with a median best tumor response of − 27.2%. Although the median progression-free survival (PFS) for the phase II part has not been reached, median PFS in the phase I part was 52 cycles, which is approximately 4 years [60].

Similar to the data described for stratum 1 of SPRINT, 25 mg/m^2^ selumetinib twice daily resulted in PN shrinkage in the majority (18 of 25 [72%]) of children with NF1-PN without clinically significant morbidity (stratum 2), and 68% had a PR lasting at least 1 year [58]. No new PN-related symptoms developed while on selumetinib, and patient-reported outcome measures (pain intensity, pain interference, QoL, and global impression of change) indicated declines in tumor-related pain intensity. These data suggest that selumetinib may prevent the development of PN-related morbidities [58].

The safety and tolerability profile of selumetinib in this pediatric population is acceptable and consistent with that reported in adult studies [49, 50, 58, 59]. Among children with symptomatic PN (stratum 1) in phase II, the most common adverse events (AEs) (all grades) with selumetinib included vomiting (80%), diarrhea (66%), acneiform skin rash (60%), nausea (60%), abdominal pain (56%), mucositis (50%), and fatigue (46%) [50]. Decreased left ventricular ejection fraction was detected in 14% of patients and edema was observed in 18% of patients; increased creatine phosphokinase was the most commonly detected laboratory abnormality [50].

Ongoing studies are aimed at expanding the potential role of selumetinib in pediatric and adult patients with NF1-PN and NF1-associated malignancies; a summary is provided in Table 3.

**Table 3:** Ongoing studies evaluating selumetinib in pediatric and adult patients with NF1-PN and NF1-associated malignancies

Trial name and identifier	Phase	Population	Aim	Location	N	Status	Estimated completion date
NCT04590235[61]	I	Pediatric and adult patients with NF1 and inoperable PN	To assess safety, tolerability, pharmacokinetics, and clinical efficacy	China	32	Active, not recruiting	October 2023
NCT04495127[62]	I	Pediatric patients with NF1 and inoperable PN	To assess safety, tolerability, pharmacokinetics, and clinical efficacy	Japan	12	Active, not recruiting	March 2023
SPRINKLE NCT05309668[63]	I/II	Pediatric patients with NF1 and inoperable PN	To define a dosing regimen and assess the pharmacokinetics and safety of the granule formulation of selumetinib	USA, Germany, Italy, Japan, the Netherlands, Russian Federation, Spain	38	Recruiting	July 2027
NCT05101148[64]	I	Adolescent patients with NF1 and inoperable PN	To assess the effect of food on the pharmacokinetics and gastrointestinal toxicity of selumetinib	USA, Poland, Russian Federation, Spain	24	Active, not recruiting	March 2023
NCT02407405[65]	II	Adult patients with NF1 and inoperable PN	To assess efficacy and safety	USA	36	Active, not recruiting	January 2025
KOMET NCT04924608[66]	III	Adult patients with NF1 and inoperable PN	To assess efficacy and safety	Global	146	Recruiting	May 2025

*NF1* Neurofibromatosis type 1, *PN* Plexiform neurofibromas, *USA* United States of America

### Investigational MEK inhibitors

#### Binimetinib

Binimetinib is an oral, selective, MEK1/2 inhibitor approved for the treatment of patients (adult and pediatric) with unresectable or metastatic melanoma, naïve to BRAF-inhibitor treatment, with *BRAF* V600E or V600K mutations [67]. Binimetinib is currently being evaluated in a phase II trial in pediatric and adult patients aged ≥ 1 year with inoperable NF1-PN (NCT03231306) [68]. Preliminary data are available for 20 adults, 13 (65%) of whom achieved a PR (≥ 20% decrease in NF1-PN volume) (Table 2) [55]. The starting dose was originally 45 mg twice daily; however, the dose protocol was amended and the dose lowered to 30 mg twice daily because of intolerable Grade 2/3 toxicities in five of the first 12 patients. Nine of 11 patients who initiated treatment on the lower dosage achieved a PR by cycle 12. Preliminary data suggest that binimetinib is reasonably well tolerated at 30 mg twice daily; treatment-related Grade 3 toxicities included rash (8%), nausea (4%), and fatigue (4%) [55].

#### Mirdametinib

Mirdametinib is an orally administered investigational MEK1/2 inhibitor that has been evaluated in an open-label phase II trial (NCT02096471) involving patients with NF1-PN aged ≥ 16 years who received mirdametinib 2 mg/m^2^ (max 4 mg) twice daily in 4-week cycles (3 weeks on/1 week off). PRs were achieved in eight of 19 patients (42%); a further 10 patients had stable disease and one experienced progression (defined as a ≥ 20% increase in PN volume, ≥ 13% increase in the product of the two longest perpendicular diameters, or ≥ 6% increase in the longest diameter) (Table 2) [51]. No patients discontinued treatment because of dose-limiting toxicity, one patient experienced Grade 3 treatment-related back and abdominal pain, and the most common treatment-related AE was acneiform rash (94.7%) [51]– one of the most frequent dermatologic toxicities associated with MEK inhibitors (trametinib, cobimetinib, binimetinib, selumetinib), especially when they are used as monotherapy [69].

Results are available from 20 adults enrolled in an ongoing phase II clinical trial (ReNeu, NCT03962543) of mirdametinib in patients with progressive or symptomatic NF1-PN causing significant morbidity [52]. After a median duration of 10 months, 16 of 20 patients remain on treatment. The overall response rate was 50%, with six of seven PRs confirmed on subsequent assessments. Treatment with mirdametinib was also associated with reductions in pain and significant improvements in QoL in patients with a PR [52]. As expected with this class of targeted therapy, the most common AEs reported were rash, nausea, and diarrhea (one patient experienced Grade 3 rash) [52].

#### Trametinib

Trametinib is an oral, selective, MEK1/2 inhibitor that is approved for the treatment of patients (pediatric and adults) with melanoma, non-small cell lung cancer, and anaplastic thyroid cancer with *BRAF* V600E or V600K mutations [70].

Trametinib has been investigated in a phase I/IIa trial (NCT02124772) in patients aged 1–17 years with medically significant NF1-PN. Trametinib was administered once daily at a dose of 0.032 mg/kg (aged ≤ 5 years) or 0.025 mg/kg (aged ≥ 6 years) with a maximum daily dose of 2 mg/day [71]. Preliminary data show that 12 of 26 patients (46%) achieved a PR (Table 2) [54]. In line with the expected safety profile of MEK inhibitors, the most common AEs were paronychia (50%) and rash (40%) [54].

### Investigational multiple tyrosine kinase inhibitor

#### Cabozantinib

Cabozantinib is an oral tyrosine kinase inhibitor with known targets such as MET and vascular endothelial growth factor receptor 2, among others [72]. Cabozantinib is approved for the treatment of adult patients with hepatocellular carcinoma or advanced renal cell carcinoma [72]. However, the safety and efficacy of cabozantinib in children and adolescents aged < 18 years have not yet been established [72]. In preclinical studies, cabozantinib reduced the number of PN, PN volume, and PN angiogenesis when compared with vehicle control in *NF1* mutant mice [53]. In a phase II clinical study (NCT02101736) in patients aged ≥ 16 years with unresectable progressive or symptomatic NF1-PN, cabozantinib 40–60 mg once daily led to a PR in eight of 19 patients (42%) (Table 2) [53]. Achievement of a PR was associated with significant reductions in PN pain intensity and pain interference in daily life [53]. Two patients discontinued treatment for dose-limiting toxicities (palmar-plantar erythrodysesthesia) [53]. In line with the expected safety profile of MEK inhibitors, common AEs included gastrointestinal events, hypothyroidism, fatigue, and palmar plantar erythrodysesthesia [53].

## Clinical decision making

Not all PN require intervention and there is no single treatment pathway for patients with NF1-PN; watchful waiting, surgery, medical treatment, or a combination of these modalities are all possible options [30]. Treatment should be individualized based on recommendations from the MDT, including consideration of the size and location of the PN, growth trajectory, effects on adjacent tissues, and current or potential complications while taking into account patient and family preferences [29, 30]. Many factors influence treatment decisions, such as the age of the patient, the severity of symptoms and the presence of NF1-related comorbidities, and the possibility of developing severe and irreversible complications if PN continue to grow [30]. These factors affect decisions regarding the suitability and preference for observation versus treatment and, when treatment is preferred, for surgical versus medical treatment [30]. In addition, it may be beneficial for patients to travel to specialist facilities for diagnosis and treatment; however, we recognize that frequent, long-distance travel is not always feasible. Therefore, a combination of local toxicity evaluations, such as blood tests, telemedicine visits, and attempts to collaborate with local providers could be used once a care plan has been determined. This co-management of patients may improve as awareness of the diagnosis and management of NF1 increases [29].

### Surgery

As described above, complete resection and debulking surgery are important treatment strategies for PN, having the advantage of providing immediate relief from large and/or painful PN, whereas medical therapy typically takes longer to provide relief [73].

Debulking surgery, when complete resection is not feasible, is typically directed at large PN that may be impinging on vital structures including the airway or spinal cord or at improving PN-related disfigurement or organ function, such as renal function when there is obstructive hydronephrosis. As such, PN location and structural characteristics are important considerations prior to surgical resection. Surgery is inherently more challenging when a PN involves structures located in the head or neck, mediastinum, or deep pelvis. PN that involve the brachial or lumbar plexus often affect motor function, cause pain, and require specialized expertise for surgical debulking or excision. Common indications for surgical resection include neurologic dysfunction, pain, airway difficulties, disfigurement, orthopedic issues, the need for diagnostic biopsies, or pre-malignancy [24, 36, 74]. NF1-PN are typically highly vascular and blood loss may limit the surgical procedure; hence, in our experience, debulking of large superficial NF1-PN is often staged with multiple surgeries [42, 75]. Pre-operative embolization can be used to mitigate the inherent risk of hemorrhage due to the vascularity of NF1-PN, which is more frequent with large neurofibromas located in anatomic regions where a tourniquet cannot be applied, which can lead to major surgical morbidity [41, 42]. Furthermore, once there is a suggestion of malignant or pre-malignant degeneration in a PN (evaluated by pre-operative biopsy or imaging), ^18^FDG PET MRI or CT combined with regional MRI may be advised and timely surgical resection of the targeted area is warranted [76–78]. NF1-PN debulking requires the surgeon to constantly balance the risks of neurologic deficit, bleeding, and potential compromise of adjacent structures with the benefit of resection. Intra-operative clinical decision making is critical to achieve this balance.

Because the primary goal of treatment is to improve or prevent PN-associated morbidity, predicted outcome is an important consideration when selecting a surgical treatment option. Surgical outcomes can be variable and a proportion of patients who undergo surgery may experience either no change in PN-related symptoms or only partial resolution [75]. In addition, PN regrowth after surgery is not uncommon, especially in patients younger than 21 years of age [24, 74, 75]. Indeed, rates of PN regrowth after partial resection have been reported to range from 29 to 68% depending on the extent of resection and can be 20% after complete excision, although confirming complete resection can be challenging [24, 74]. Younger age in addition to tumor type, location (tumors of facial area or trunk), depth, and diffuse growth type are associated with tumor recurrence [75]. Postoperative PN regrowth has historically been a cause for concern, which led to a cautious approach regarding the surgical management of NF1-PN; however, postoperative progression of PN may not be significantly different from the natural growth behavior, suggesting that postoperative tumor growth could be unrelated to and not promoted by surgery [75].

### Medical therapy

In addition to surgery, medical therapy is now an available treatment option for NF1-PN. Data from recent MEK inhibitor clinical trials are available to guide clinical treatment decision making. However, much remains to be learned about optimal use of medical therapy and ways in which we can combine medical and surgical treatment to optimize outcomes for individual patients.

Currently, the only approved medical therapy for NF1-PN is selumetinib, which is indicated for the treatment of pediatric patients (aged ≥ 2 years in the US and aged ≥ 3 years in Europe) with NF1 who have symptomatic, inoperable PN [56, 57]. An inoperable PN was defined as a PN that could not be removed completely by surgery without risk of substantial morbidity, or if the patient or family refused a surgical approach [79]. Examples of symptomatic PN include those with associated pain, disfigurement, and functional impairment. In contrast, another population to consider are those with PN that although not currently associated with morbidity pose a risk of future tumor-related complications [49, 50]. Examples include head and neck PN that could compromise the airway or great vessels or brachial or lumbar plexus PN that could cause nerve compression and loss of function [58].

Although treatment with a MEK inhibitor is indicated in patients with inoperable, symptomatic NF1-PN, there are data to suggest that treatment of patients with inoperable PN not currently causing clinically significant morbidity but deemed at risk for developing serious PN-related complications may be effective in preventing PN growth and PN-related morbidity [80, 81]. Indeed, in SPRINT, patients with no significant PN-related morbidity at enrollment but the potential for development of PN morbidity (stratum 2) demonstrated a PR rate similar to patients with PN-related morbidity at enrollment (stratum 1) (68% vs. 71%) [50, 58]. Therefore, further research is warranted to determine whether it is beneficial to initiate treatment prior to the onset of symptoms, thereby showing the effectiveness of reducing PN volume and preventing PN from becoming symptomatic, particularly in selected at-risk patients with rapidly progressing PN.

Treatment with targeted therapy also has the potential to facilitate multimodal therapy for large inoperable PN and to achieve better clinical response or time to progression [46]. A case report describes an 11-year-old girl with NF1 in whom extensive growth of cervical PN masses rendered the cervical column inaccessible to recommended surgical intervention to prevent paraplegia. Treatment with trametinib initiated with a single 0.05 mg (0.015 mg/kg) dose, increased after 1 week to 0.5 mg (0.03 mg/kg) twice daily for 6 months, resulted in a 22% reduction in tumor volume, which was sufficient to enable surgery [82]. Prospective studies of pre- and post-operative MEK inhibition are required to develop and validate a multimodal treatment algorithm and to more comprehensively understand how medical therapy and surgical approaches may augment each other in the treatment of NF1-PN. Furthermore, it has been proposed that by understanding the mechanism of response for NF1-PN, the development of rational combinations of MEK inhibitors with other targeted or cytotoxic therapies may be possible [83].

Medical therapy may also play a role in patients with PN that are associated with more severe symptoms and/or causing substantial morbidity; for example, organ dysfunction such as hydronephrosis, airway compression, or sensory dysfunction from a head or neck PN [18, 30, 84]. Among pediatric and adult patients with NF1-PN and spinal neurofibromas (associated with pain numbness, paresthesia, motor weakness, or gait abnormalities), treatment with selumetinib (12 cycles at the recommended dose of 25 mg/m^2^ twice daily) was associated with a reduction in spinal neurofibroma burden and associated improvements in spinal canal distortion, circumferential cerebrospinal fluid disruption, and spinal cord deformity [85].

Another key consideration in clinical decision making is that younger patients appear to be more likely to benefit from early initiation of a MEK inhibitor. Rapidly growing PN tend to be observed in younger children (i.e., aged ≤ 5 years), and progressive PN (those that grow by ≥ 20% per year) are unusual after adolescence [17, 19, 86]. An analysis of data from the SPRINT trial showed that children who achieved a PR were slightly younger (median age 9.5 years) than those who did not (median age 13.3 years); however, age did not correlate with maximal PN shrinkage in patients who achieved a PR [80]. Clinical experience suggests that younger patients tolerate medical therapy better and have better medication adherence than adolescents [80]. However, it is important to note that selumetinib is currently dosed in a fasted state, which may be considered a limitation for some [56]. These potential advantages of early initiation of medical therapy must be balanced against a lack of long-term data regarding the effect of MEK inhibition on growth and development and whether medical therapy changes the natural history of PN.

The optimal duration of therapy with a MEK inhibitor is still unknown. In the SPRINT trial, a median of eight cycles or 6.9 months of treatment with selumetinib was required before evidence of a PR became apparent [80]. This is similar to the time to response observed with other MEK inhibitors such as binimetinib (12 cycles) [55]. However, in SPRINT, symptomatic benefit often occurred before or in the absence of radiological benefit [50]. It is as yet unclear how long treatment must be continued to sustain clinical benefit for these patients. The results of natural history studies of the growth of PN suggest that extended treatment may be required [73]. The median duration of response was not reached in the phase II part of SPRINT; however, 82% of patients with a confirmed PR had a duration of response of at least 12 months, and the 3-year PFS was 84% [50]. Prospective studies are required to determine whether it is possible to discontinue MEK inhibitor treatment once growth of PN has slowed or stopped in late adolescence or in young adulthood [73]. It is also important to consider the safety profile of MEK inhibitors when considering longer-term treatment; careful monitoring and management of AEs is critical (Table 4).

**Table 4:** Monitoring and management of significant adverse events associated with selumetinib therapy [56]

Adverse event	Incidence in SPRINT	Recommended monitoring	Recommended management
Rash	91%	Monitor for rash at each encounter	Withhold treatment, reduce dose, or discontinue selumetinib
Diarrhea	77%	Monitor for diarrhea at each encounter	Loperamide Increase fluid intake Withhold treatment, reduce dose, or discontinue selumetinib
Increased CPK	76%	Measure CPK at baseline	Evaluate patients for rhabdomyolysis if CPK is increased Withhold treatment, reduce dose, or discontinue selumetinib
LVEF ≥ 10% below baseline (and below institutional lower limit)	23%	Echocardiogram at baseline, q3 months during the first year, and q6 months thereafter	Perform echocardiogram Withhold treatment, reduce dose, or discontinue selumetinib
Ocular toxicity (blurred vision, photophobia, cataracts, and ocular hypertension)	15%	Ophthalmic exam at baseline, and at regular intervals thereafter Optical coherence tomography q3 weeks until resolution in patients with RPED	Perform ophthalmic exam Withhold treatment, reduce dose, or discontinue selumetinib depending on severity Withhold selumetinib in patients with RPED, resume once resolved Discontinue selumetinib in patients with RVO

*CPK* Creatine phosphokinase, *LVEF* Left ventricular ejection fraction, *q3* every 3, *q6* every 6, *RPED* Retinal pigment epithelial detachment, *RVO* Retinal vein occlusion

It is important to note that clinical trials use sophisticated volumetric analysis that may not be available at all institutions. In a real-world clinical setting, radiologic progression may be monitored with standard MRI techniques; moreover, symptomatic improvement may be preferred to radiologic measurements to monitor the efficacy of treatment. Indeed, radiologic responses alone are not considered to be sufficient evidence of efficacy by the US Food and Drug Administration (FDA). The SPRINT trial was designed to show that reductions in PN volume (objective responses) were accompanied by detectable clinical improvements in PN as reflected by reductions in functional impairment, symptoms, or disfigurement, and improvement in QoL [87]. It is also important to understand and not be discouraged when there is no measurable PN shrinkage using standard MRI, which can be less sensitive at detecting changes in PN volume compared with volumetric MRI.

## Future directions

With increasing research into targeted therapies, a new era has arrived. Selumetinib has shown promising efficacy as a non-surgical option for children with NF1-PN, with a demonstrated ability to shrink PN and improve patient-reported outcomes alongside an acceptable tolerability profile [49, 50, 58]. The availability of medical therapy as another treatment option to surgery introduces additional dimensions in clinical decision making and management considerations.

However, there are many questions that remain to be answered regarding the use of selumetinib and other agents in development in pediatric patients with NF1-PN. These include alternative dosing formulations (e.g., intermittent, non-continuous dosing [83]) to improve dosing precision for younger pediatric patients, ideal treatment duration, predictors of response, and optimal sequencing with surgery. In addition, large, long-term studies are required to better define the efficacy and safety of these agents in this population and to determine the extent to which treatment modifies the natural history of this disease. Better understanding of which patient characteristics (age, sex, molecular biology, genetics, PN size and location, etc.) predict response would help optimize treatment. Prospective data are also required to further define the role of surgery in this new medical therapy era, the optimal sequence of surgery and medical therapy in different patient subgroups (i.e., pre-operative and post-operative use), and to determine the potential for other MEK inhibitors in treatment-resistant patients.

The potential impact of long-term medical therapies such as MEK inhibitors on development, growth, and cognition is currently unknown and are particularly important considering that patients referred for NF1-PN treatment are children. Monitoring parameters and the appropriate duration of follow-up after treatment discontinuation also remain to be determined.

In addition to NF1-PN, there are ongoing studies to further investigate the efficacy of MEK inhibitors in other NF1-related tumors, including cutaneous neurofibromas and low-grade glioma.

In conclusion, it is clear that clinical decision making is complex for children with NF1-PN [1, 30]. Although surgery is a valuable management strategy and remains a standard treatment option for many patients, it can be complicated, is not always feasible, and is associated with inherent risks and a likelihood of recurrence [1, 30]. A recently approved medical therapy option has demonstrated promising efficacy with significant shrinkage of PN, sustained PRs, and improvements in patient-reported outcomes in pediatric patients with symptomatic, inoperable NF1-PN. Based on the current evidence, careful consideration and balance of risk/benefit are still required when considering treatment options for NF1-PN, and ongoing research will better define the role of MEK inhibitors in the overall management of children with this condition.

## Data Availability

Not applicable.

## Abbreviations

AE: Adverse event
CPK: Creatine phosphokinase
CT: Computed tomography
ENT: Ear nose and throat
EU: European Union
FDA: US Food and Drug Administration
FDG-PET: Fluorodeoxyglucose positron emission tomography
GI: Gastrointestinal
LVEF: Left ventricular ejection fraction
MAPK: Mitogen-activated protein kinase
MDT: Multidisciplinary team
MEK: Mitogen-activated protein kinase
MRI: Magnetic resonance imaging
NF1: Neurofibromatosis type 1
ORR: Objective response rate
PFS: Progression-free survival
PN: Plexiform neurofibromas
PR: Partial response
q3: every 3
q6: every 6
QoL: Quality of life
RPED: Retinal pigment epithelial detachment
RVO: Retinal vein occlusion
USA: United States of America
